# Supplementary material for: Impact of Fenugreek on Milk Production in Rodent Models of Lactation Challenge
Source: Nutrients. 2019 Oct 24;11(11):2571. doi: 10.3390/nu11112571 (PMC6893785; doi:10.3390/nu11112571)
Supplement: Supplementary file 1 [file nutrients-11-02571-s001.zip › Table S1.docx]

| Composition (g/100g dry matter) | Experimental diets | | | |
| --- | --- | --- | --- | --- |
|  | 20% protein | 20% protein + fenugreek | 8% protein | 8% protein + fenugreek |
| Cellulose | 5.00 | 4.97 | 5.00 | 4.96 |
| HCl Casein | 20.00 | 19.87 | 8.00 | 7.94 |
| L Cystine | 0.30 | 0.30 | 0.30 | 0.30 |
| AIN93G Mineral Mix | 3.50 | 3.48 | 3.50 | 3.47 |
| AIN93Vx Vitamin Mix | 1.00 | 0.99 | 1.00 | 0.99 |
| Choline bitartrate | 0.25 | 0.25 | 0.25 | 0.25 |
| TBHQ | 0.0014 | 0.0014 | 0.0014 | 0.0014 |
| Maltodextrin | 13.20 | 13.11 | 15.72 | 15.60 |
| Corn starch | 39.75 | 39.48 | 47.32 | 46.94 |
| Granular sugar | 10.00 | 9.93 | 11.91 | 11.82 |
| Corn oil | 7.00 | 6.95 | 7.00 | 6.94 |
| Fenugreek seed dry extract | 0.00 | 0.67 | 0.00 | 0.79 |
| **Characteristics** |  | | | |
| Moisture (g/100g diet) | 7.0 | 7.0 | 7.0 | 7.0 |
| Energy (kcal/g) | 3.76 | 3.73 | 3.76 | 3.73 |
| Trigonelline content (µg/g diet) | 0.1 | 12.5 | 0.2 | 14.9 |

Table S1: Composition and characteristics of the experimental based on AIN-93G diets.
