# Supplementary material for: Impact of Fenugreek on Milk Production in Rodent Models of Lactation Challenge
Source: Nutrients. 2019 Oct 24;11(11):2571. doi: 10.3390/nu11112571 (PMC6893785; doi:10.3390/nu11112571)
Supplement: Supplementary file 1 [file nutrients-11-02571-s001.zip › Figure S1.docx]

Figure S1 : Time course of D_2_O concentrations in dam’s plasma
