# Supplementary material for: Impact of Fenugreek on Milk Production in Rodent Models of Lactation Challenge
Source: Nutrients. 2019 Oct 24;11(11):2571. doi: 10.3390/nu11112571 (PMC6893785; doi:10.3390/nu11112571)
Supplement: Supplementary file 1 [file nutrients-11-02571-s001.zip › Table S2.docx]

| Lactation challenges | **Control** | **12 pups per litter** | | | | **8% protein perinatal diet** | |
| --- | --- | --- | --- | --- | --- | --- | --- |
| Groups | **NP:8** | | **NP:12** | **NPF:12** | **LP:8** | | **LPF:8** |
| Observations, n | 7 | | 10 | 10 | 8 | | 9 |
| **TBWd**, g | 245.4 ± 8.2 | | 240.4 ± 7.4 | 261.9 ± 7.7 | 225.8 ± 9.1 | | 230.8 ± 3.1 |
| **TBWd**, % | 72.9 ± 0.8 | | 71.9 ± 0.8 | 73.5 ± 0.8 | 72.0 ± 1.3 | | 72.4 ± 0.9 |
| Observations, n | 8 | | 11 | 11 | 8 | | 9 |
| **K31**, h^-1^.10^-3^ | 3.92 ± 0.12 | | 4.64 ± 0.14 | 5.00 ± 0.17 | 2.46 ± 0.10 | | 2.38 ± 0.12 |
| FSD, % | 2.08 ± 0.21 | | 2.83 ± 0.38 | 2.64 ± 0.27 | 4.07 ± 0.75 | | 2.60 ± 0.47 |
| **K42**, h^-1^.10^-3^ | 3.86 ± 0.13 | | 4.81 ± 0.16 | 5.01 ± 0.15 | 2.48 ± 0.10 | | 2.42 ± 0.11 |
| FSD, % | 2.42 ± 0.34 | | 3.09 ± 0.42 | 2.77 ± 0.28 | 3.61 ± 0.47 | | 2.56 ± 0.50 |

Table S2: Model coefficients for measurement of milk flow by water turnover method.

TBWd correspond to the total body water of dams in g or based on dams’ weight (%). K31 and K42 are the output flow constants from dam to its male litter and from dam to its female litter respectively. FSD is calculated for each individual and corresponds to the accuracy in determination of the flow constants with the fitting model. Milk flow is calculated by multiplying flow constant by TBWd in g. Values are mean ± SEM for each group.
