# Supplementary material for: Impact of Fenugreek on Milk Production in Rodent Models of Lactation Challenge
Source: Nutrients. 2019 Oct 24;11(11):2571. doi: 10.3390/nu11112571 (PMC6893785; doi:10.3390/nu11112571)
Supplement: Supplementary file 1 [file nutrients-11-02571-s001.zip › Figure S2.docx]

Figure S2: Effect of fenugreek on macronutrient consumption by pups in the litter size challenge
